# An efficacy and safety study of rivaroxaban for the prevention of deep vein thrombosis in patients with left iliac vein compression treated with stent implantation (PLICTS): study protocol for a prospective randomized controlled trial

**DOI:** 10.1186/s13063-020-04742-z

**Published:** 2020-09-29

**Authors:** Miaomiao Li, Libin Zhang, Kaijie Zhang, Yuefeng Zhu, Zhenyu Shi, Wan Zhang, Bin Gao, Lubin Li, Zhengdong Fang, Li Yin, Bing Chen, Zhenjie Liu

**Affiliations:** 1 Department of Vascular Surgery, The Second Affiliated Hospital of Zhejiang University, School of Medicine, Hangzhou, 310052 China; 2 School of Medicine, Zhejiang University, Hangzhou, 310009 Zhejiang Province China; 3 Department of Cardiology, Sir Run Run Shaw Hospital of Zhejiang University, School of Medicine, Hangzhou, 310016 China; 4 Department of Vascular Surgery, Sir Run Run Shaw Hospital of Zhejiang University, School of Medicine, Hangzhou, 310016 China; 5 Department of Vascular Surgery, Zhongshan Hospital of Fudan University, Shanghai, 210023 China; 6 Department of Vascular Surgery, Huadong Hospital of Fudan University, Shanghai, 210023 China; 7 Department of Vascular Surgery, The Fifth People’s Hospital of Shanghai, Fudan University, Shanghai, 200240 China; 8 Department of Vascular Surgery, Yantai Yuhuangding Hospital, Yantai, 264000 China; 9 Department of Vascular Surgery, The First Affiliated Hospital of USTC, Division of Life Sciences and Medicine, University of Science and Technology of China, Hefei, 230001 Anhui People’s Republic of China

**Keywords:** Left iliac vein compression syndrome, Thrombotic, Warfarin, Rivaroxaban, Stent implantation

## Abstract

**Background:**

Balloon dilatation with stent implantation has been proved to be an effective option for left iliac vein compression syndrome (LIVCS), but thrombosis may still occur after the operation. Currently, warfarin is used for anticoagulant therapy, but long-term monitoring is required, while rivaroxaban does not need laboratory monitoring, which can simplify treatment. Therefore, this study aimed to compare the efficacy and safety of rivaroxaban and warfarin in anticoagulation.

**Methods:**

This study is a multicenter, randomized controlled trial. We will recruit 224 patients with thrombotic LIVCS from 9 hospitals. Moreover, these patients will be randomized to either the experimental group (rivaroxaban) or the control group (warfarin plus nadroparin). The primary outcome is stent occlusion rate. Secondary outcomes are quality of life scale survey results, all-cause mortality, anticoagulation-related mortality, and the proportion of participants with stent displacement/fracture, thrombosis, hemorrhage, and other vascular events.

**Discussion:**

This study will provide reliable, evidence-based clinical evidence for the efficacy and safety of rivaroxaban antithrombotic therapy after stent implantation.

**Trial registration:**

ClinicalTrials.gov NCT04067505. Registered on August 26, 2019.

## Background

Left iliac vein compression syndrome (LIVCS) is a disease of iliac vein stenosis/occlusion caused by chronic friction and compression of the left iliac vein by the right common iliac artery and lumbar vertebra, which results in blood flow disorders in lower limbs and pelvic veins. It is also called Cockett syndrome [1] or May-Thurner syndrome [2]. Left iliac vein stenosis or occlusion can cause a deep venous thrombosis, and chronic venous insufficiency of the left lower extremity, manifesting as swelling, pain, varicose veins, skin pigmentation, skin dystrophy, and venous ulceration in severe cases, affect the quality of life and labor capacity. With the growing awareness of this disease and the development of various imaging examinations, the detection rate of LIVCS has gradually increased [3, 4]. The incidence of LIVCS was about 14.8% in patients with uncomplicated venous insufficiency of the left lower extremity [5]. Moreover, 50 to 60% of patients with left iliac vein thrombosis have left iliac vein compression [6]. Recently, left iliac vein balloon dilatation with stent implantation has been used to treat patients with LIVCS and have achieved good results [7, 8].

Interventional treatment of iliac vein stenosis or occlusion can directly cause local trauma and intimal injury, which is a clear inducement of local thrombosis. Therefore, high-intensity anticoagulation therapy is still needed to prevent secondary thrombosis in a stent after left iliac vein balloon dilatation with stent implantation. At present, the postoperative anticoagulation regimen of these patients is early heparin anticoagulant therapy and later warfarin anticoagulant therapy. Warfarin is a standard oral anticoagulant for a long time, with good anticoagulant effect and low price. However, due to the narrow therapeutic window of the drug, patients need to adjust the dosage according to the coagulation function under the guidance of doctors. However, doctors at grass-roots level are not familiar with the use of warfarin, coupled with patients’ lack of awareness of warfarin, resulting in more difficult follow-up management, the uncertain treatment effect of warfarin, and even severe bleeding complications [9]. On the one hand, according to relevant research data, the incidence of warfarin-related bleeding is about 1–2% [9], among which intracranial hemorrhage is about 0.76% per year, which is the most worrying bleeding complication [10]. On the other hand, due to the need for repeated visits, some patients automatically give up treatment, resulting in recurrence or aggravation of thrombosis.

Rivaroxaban is a direct oral anticoagulants (DOACs) that function as a factor Xa inhibitor. It is more safe and can simplify treatment. It is also not easy to interact with food or drugs. Previous studies have shown that rivaroxaban is effective in preventing deep venous thrombosis after orthopedic surgery [11, 12]. Rivaroxaban has also been reported to be safe and effective in anticoagulation therapy for patients with deep venous thrombosis and pulmonary embolism, and laboratory monitoring is not required [13–15], while due to the limitation of indications, DOAC is mainly used for the prevention and treatment of venous thrombosis. Rivaroxaban lacks sufficient clinical data for adjuvant anticoagulation therapy after balloon dilatation with stent implantation. Therefore, we design this study to access the efficacy and safety of rivaroxaban for the prevention of deep vein thrombosis in patients with left iliac vein compression treated with stent implantation.

For the postoperative oral anticoagulation regimen for patients with LIVCS, this study will evaluate the efficacy and safety of rivaroxaban after stent implantation in the left iliac vein through a prospective, randomized controlled trial. Also, this study will provide the basis for LIVCS treatment guidelines and explore the clinical indications of rivaroxaban.

## Methods

### Study design and setting

**Fig. 1:**
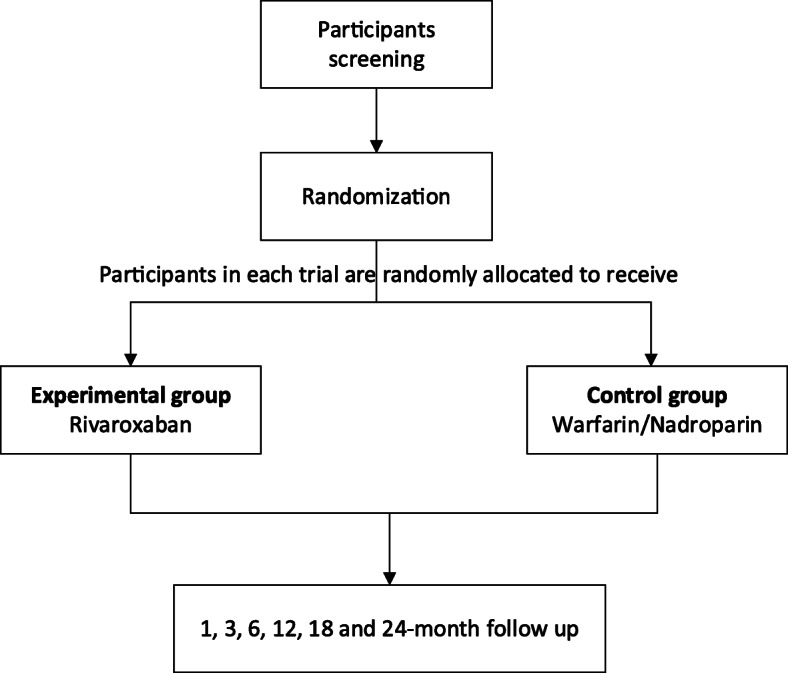
The procedure of screening, randomization, and follow-up of patients in the trial

The study is a prospective, randomized, multicenter, controlled trial that will evaluate the efficacy and safety of rivaroxaban for the prevention of deep vein thrombosis in patients with left iliac vein compression syndrome treated with stent implantation. The ethics committee approved the study at each participating hospital and written informed consent will be obtained from patients before enrolment. Doctors in charge of each center is responsible for this work. The independent adjudication committee blinded to the intervention will adjudicate all events. We will randomly allocate patients to either the experimental group or the control group after enrollment. The study was registered in the website of ClinicalTrials.gov (registration no. NCT04067505). And the study design is described in Fig. 1.

### Sites and patients

Before the start of this study, a total of 9 hospitals participating in the trial fully communicated to determine the clinical diagnosis and treatment procedure of LIVCS according to the existing clinical guidelines or routines. Moreover, the anticoagulation treatment and monitoring program after stent implantation were also formulated. The diagnosis and treatment process, diagnostic criteria, and research protocols were published and unified training at the kick-off meeting.

For this study, we will recruit patients aged 18 to 75 with LIVCS from 9 sites in China. The diagnostic criteria of LIVCS (all need to meet the following three standards) are as follows: (1) definite clinical manifestations of deep venous thrombosis or venous insufficiency of the left lower extremity, such as swelling, pain, heaviness, skin dystrophies, venous ulcers; (2) the visualization of a > 50% reduction in the luminal diameter of the vein according to color Doppler ultrasound (DUS) and computed tomography venography (CTV); (3) the formation of collateral circulation; and (4) a pressure gradient of > 2 mmHg across the stenosis while the patient was in a supine position [5]. According to the clinical diagnosis and treatment process of LIVCS, the participating hospitals will perform left iliac vein color Doppler ultrasound and left iliac vein CTV screening for patients with the above-mentioned symptoms. Patients in accordance with the diagnostic criteria will be diagnosed as LIVCS. Patients with a definite diagnosis of left iliac vein compression syndrome who conform to the following inclusion and exclusion criteria will be included in the study.

The inclusion criteria are as follows: patients with thrombotic LIVCS who underwent left iliac vein stent implantation. Patients with following conditions will be excluded: age < 18 years or age > 75 years; with a history of pelvic surgery, left iliac vein trauma or pelvic radiotherapy; with obvious contraindications for anticoagulation therapy; allergic to iodine contrast agents in the past; with concomitant diseases that need high-intensity anticoagulation; the anticoagulation intensity is higher than that of the patients with iliac vein therapy alone; active bleeding or potential bleeding risk; pregnant or breastfeeding women; with pelvic tumors causing compression of left iliac vein; with chronic venous insufficiency of lower extremities caused by K-T syndrome; with malignant tumors and life expectancy < 1 year; and taking CYP-450 3A4 inhibitors or inducers.

### Randomization and blinding

Blocked randomization was adopted. We will conduct central randomization based on the research electronic data capture (REDCap) system. REDCap system was developed by Paul Harris of Vanderbilt University [16]. A randomization module is put in this system. So, someone who is not involved in this study will generate the allocation sequence by REDCap system, in order to ensure allocation concealment. Then, patients will be randomly assigned to either the experimental group or the control group. It is an open-label study, while assessors will be blinded after assignment to interventions.

### Treatment/protocol of therapy

Inclusive patients will receive left iliac vein stent implantation. Participants in the experimental group will receive rivaroxaban 15 mg twice daily for 3 weeks, then 20 mg oral once daily until 6 months after the operation. Moreover, participants in the control group will receive subcutaneous nadroparin, 1.0 mg per kilogram of body weight twice daily, plus warfarin 3 mg oral once daily for 5 days after the operation, and later warfarin (oral) at individually titrated doses (0.75 mg to 18 mg) to achieve a target international normalized ratio (INR) of 2.0 to 3.0, once-daily until 6 months.

### Criteria for discontinuing or modifying allocated interventions

The standards for warfarin withdrawal or reduction were described previously [17] (Supplementary Appendix). Patients in the experimental group should stop using rivaroxaban when they have life-threatening bleeding.

Participants should withdraw from this clinical trial when they have active bleeding after anticoagulant administration, diseases that cannot continue anticoagulation, or surgery which need to stop anticoagulation. The detailed definitions for bleeding were described in Supplementary Appendix [18]. We will purchase insurance for all participants to cover study-relate adverse events.

### Surveillance and follow-up

**Fig. 2:**
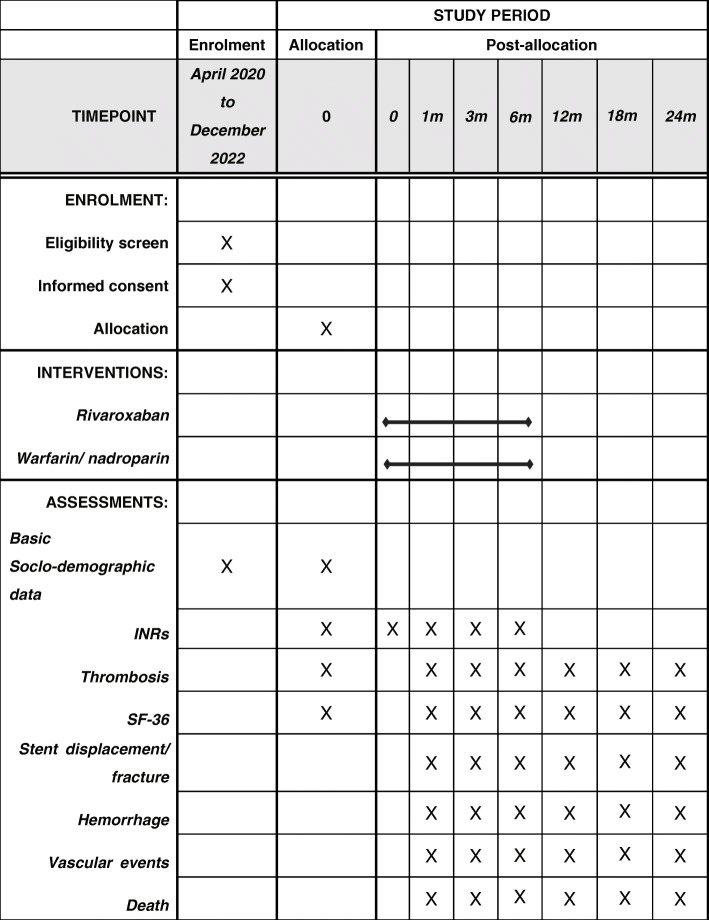
Schedule of enrolment, interventions, and assessments

All participating hospitals use the same electronic case report form (eCRF) available at REDCap system to collect data of patients. All centers participating in the trial, ethics committee, and statisticians will have access to the system. Patients’ identity information is encrypted to prevent leakage. An independent data monitoring committee is responsible for summarizing and checking the data of each center. Moreover, if they have any questions, they can contact the co-investigators of each center to review or revise. Both the rivaroxaban group and the control group will be followed at 1, 3, 6, 12, 18, and 24 months after the operation. Once bleeding, thromboembolism, or other adverse events occur, patients need to report to the hospital immediately. The schedule of enrolment, interventions, and assessments is shown in Fig. 2.

### Outcome measures

The primary outcome of the study is stent occlusion rate. The occlusion of stent will be confirmed by DUS and CTV. Stent occlusion is defined as DS > 50% for each modality with no procedure performed on the treated segment [19]. The secondary outcomes consist of quality of life scale survey results, all-cause mortality, anticoagulation-related mortality, and the proportion of participants with stent displacement/fracture, thrombosis, hemorrhage, and other vascular events. Quality of life will be assessed by MOS item short-form health survey scale (SF-36). SF-36 includes eight subscales: physical functioning (PF, ten items), role limitations due to physical health problems (RL-P, four items), bodily pain (BP, two items), general health (GH, five items), vitality (V, four items), social functioning (SF, two items), role limitations due to emotional problems (RL-E, three items), and mental health (MH, five items) [20]. Computed tomography (CT) will be used to detect stent displacement/fracture. Diagnosis of thrombosis will be based on color Doppler ultrasound and D-Dimer. Hemorrhage includes hemorrhagic stroke, gastrointestinal bleeding, hematuria, mucocutaneous hemorrhage, and another visceral bleeding. The diagnosis of hemorrhage will be based on clinical history, symptoms, laboratory examination, and imaging findings. For example, hematuria will be diagnosed according to the results of urinalysis. Moreover, the diagnosis of vascular events, including acute coronary syndrome, ischemic stroke, pulmonary embolism, transient ischemic stroke, and other parts of vascular embolism, will depend on CT/MRI.

### Sample size calculation

According to Snyder et al., stent occlusion rate of thrombotic LIVCS was 20.2% [21]. For the noninferiority test of group design, 202 cases recruited in the study are enough under the power of 0.8, two-sided alpha level of 0.05, and the non-inferiority margin of 0.1. We will include 224 samples in the study, taking into account a dropout rate of 10%, 112 in the experimental group, and 112 in the control group.

### Statistical analysis

We will perform statistical analysis on an intention-to-treat (ITT) basis. All data will be reported as mean ± standard deviation or numerical value (percentage). Non-normal distribution data will be presented as median (quartile interval, IQR). The chi-square test will be used to compare the categorical variables between the two groups. Continuous variables will be compared using sample *t* test for normally distributed data and Mann-Whitney *U* test for continuous variables of abnormal distribution. A non-corrected chi-square test will first compare the outcome variables of binary classification, and then the Cox proportional hazards model will be established to correct other confounding factors. The survival differences of the patients between the two groups will be compared using Log-Rank, and Kaplan-Meier survival curves will describe the results. Statistical analysis will be performed by using GraphPad Prism, and software R. *P* < 0.05 will be considered statistically significant.

## Discussion

Stent implantation is considered to be a feasibility, safety, and effective treatment for iliac venous stenosis, demonstrating a good long-term patency rate [22–24]. While as a result of vein intima defect or direct injury, residual thrombus and the stimulus of the stent, thrombosis may be induced after stent implantation. Moreover, due to the fact that venous blood flow velocity is slower than arterial blood flow and thrombus can cause distal venous return disturbance and insufficient blood flow, it is required that high-intensity anticoagulation should be performed after operation, usually for 3 to 6 months. Currently, oral warfarin anticoagulation is commonly used after iliac vein stent implantation [5]. Direct oral anticoagulants (DOACs) are considered to be an ideal alternative to warfarin for the advantages of no need for laboratory monitoring and are not easy to interact with food and drugs. However, there is no high-level evidence-based clinical evidence for DOACs in anticoagulation regimens after iliac vein stent implantation. Therefore, it is necessary to conduct a randomized controlled trial comparing the anticoagulant effects of DOACs and warfarin after intravenous therapy. As a direct oral anticoagulant, rivaroxaban has been proved to be effective and safe in some clinical studies. Hanafy AS et al. found rivaroxaban could improve short term survival rate effectively and safely in acute HCV-related non-neoplastic PVT [25]. In addition, Westendorf J et al. discovered rivaroxaban was a cheap and straightforward oral treatment option for patients with symptomatic superficial-vein thrombosis [26]. Einstein trial also showed that rivaroxaban offered a simple approach to the treatment of venous thrombosis [13]. Therefore, rivaroxaban was chosen in the study.

There are still some problems in this study. Firstly, since this study is an open-label study, researchers and competent doctors are aware of the patients’ grouping information, so we will arrange for a third party which is unaware of the clinical treatment information to collect the outcome data in order to reduce bias. Secondly, because patients need to sign an informed consent before they are included in the study, considering that rivaroxaban has been reimbursed under the health insurance in China and the price difference between warfarin and rivaroxaban is not massive, it may affect the speed of patients’ inclusion. Thirdly, due to most of the hospitals involved in this study do not have the conditions for intravascular ultrasound, our diagnosis of LIVCS might not be precise. Given this limitation, we proposed that the diagnosis should be based on both CTV and DUS. Finally, considering that there is no consensus on antiplatelet therapy for non-thrombotic IVCS, only patients with thrombotic iliac vein compression will be included in this study. As for patients with non-thrombotic IVCS, a larger sample size is needed, which will be carried out after getting more support in the future. In conclusion, this study will provide more evidence-based clinical evidence for anticoagulation after iliac vein stent implantation.

## Trial status

Ethics approval has been granted before submission. Recruiting patients for the trial has not yet started (scheduled date—April 30, 2020). We anticipate that recruitment will be completed on December 31, 2022. The current protocol is version 3.0. Any protocol amendments will be updated in ClinicalTrials.gov.

## Supplementary information

**Additional file 1.** English translation of ethical approval document.

**Additional file 2.** SPIRIT checklist.

**Additional file 3.** Informed consent.

**Additional file 4. Supplementary Appendix.**

## Data Availability

All centers participating in the trial, ethics committee, and statisticians will have access to the dataset. Other researchers can also have access to the dataset with the approval of the ethics committee.

## Abbreviations

LIVCS: Left iliac vein compression syndrome
DUS: Color Doppler ultrasound
CTV: Computed tomography venography
SF-36: 36-item short-form health survey scale
DOACs: Direct oral anticoagulants
INR: International normalized ratio
CRF: Case report form
RL-P: Physical health problems
BP: Bodily pain
GH: General health
V: Vitality
SF: Social functioning
RL-E: Role limitations due to emotional problems
MH: Mental health
ITT: Intention-to-treat
IQR: Quartile interval
