# Supplementary material for: An efficacy and safety study of rivaroxaban for the prevention of deep vein thrombosis in patients with left iliac vein compression treated with stent implantation (PLICTS): study protocol for a prospective randomized controlled trial
Source: Trials. 2020 Sep 29;21:811. doi: 10.1186/s13063-020-04742-z (PMC7526216; doi:10.1186/s13063-020-04742-z)
Supplement: Supplementary file 1 — Additional file 1. English translation of ethical approval document. [file 13063_2020_4742_MOESM1_ESM.pdf]

**Approval Form for the Ethics committee of the Second Affiliated Hospital of Zhejiang University School Medicine**			
Project acceptance NO: 2019-223 Event acceptance NO: IR2019001124<br>Approval NO: (2019) 286			
**Project Name**	An Efficacy and Safety Study of Rivaroxaban and Warfarin for the Prevention of Deep Vein Thrombosis in Patients with Left Iliac Vein Compression Treated with Stent Implantation		
**Application Type**	Clinical research		
**Department**	Vascular surgery	Project Leader	Liu Zhenjie
**Approved Document**			
**NO.**	**File name**	**Version Number**	**Language/ Version Date**
1	Informed consent	V1.1	Chinese/2019-08-19
2	Case report form	V1.0	Chinese/2019-08-01
3	Clinical research Protocol	V1.0	Chinese/2019-08-01
4	List of Participating Hospital		Chinese
**Date of Review**	Date of first review: 2019-08-14<br>Date of this review: 2019-09-02		
**Review Result**	1. After the review by the human body ethics review committee, the review result: approved. Comments and suggestions: approved.<br>2. The study will be subject to continuous review by the ethics review committee during the study, and the frequency of continuous review is 12 months from the date of study approval.		
Signature of chairman or vice chairman: Date:<br><br>Human Research Ethics Committee of the second affiliated hospital of Zhejiang university school of medicine (Seal)			
