# Supplementary material for: An efficacy and safety study of rivaroxaban for the prevention of deep vein thrombosis in patients with left iliac vein compression treated with stent implantation (PLICTS): study protocol for a prospective randomized controlled trial
Source: Trials. 2020 Sep 29;21:811. doi: 10.1186/s13063-020-04742-z (PMC7526216; doi:10.1186/s13063-020-04742-z)
Supplement: Supplementary file 3 — Additional file 3. Informed consent. [file 13063_2020_4742_MOESM3_ESM.pdf]

AF42 浙医二院人体研究知情同意书

尊敬的患者：

我们邀请您参加一项“利伐沙班和华法令对左髂静脉压迫综合征患者支架植入术后抗凝有效性和安全性的前瞻性随机对照研究”的临床研究，在您决定是否参加这项研究之前，请仔细阅读以下内容，它可以帮助您了解该项研究以及为什么要进行这项研究，研究的程序和期限，参加研究后可能给您带来的益处、风险和不便。

以下是本项研究的介绍：

一、研究背景和研究目的

左髂静脉受压综合征（Left Iliac Vein Compression Syndrome， LIVCS）是一种由于左髂静脉受右髂总动脉和腰椎的慢性摩擦、压迫导致髂静脉狭窄\闭塞,引起下肢及盆腔静脉血回流障碍的疾病，又称为Cockett综合征或May-Thurner综合征。左髂静脉受压综合征可以继发形成左下肢深静脉血栓和慢性左下肢静脉功能不全，表现为左下肢肿胀，疼痛，静脉曲张,皮肤色素沉积、皮肤营养障碍，严重时可发生静脉性溃疡，影响生活质量及劳动能力。近年来，左髂静脉球囊扩张、支架植入术开始运用于治疗LIVCS患者，并取得了良好的效果，但是介入治疗直接造成局部创伤和内膜损伤，是局部血栓形成的明确诱因。髂静脉支架植入暴露于血流中，表面无血管内皮保护，易直接形成血栓。特别是在局部血栓基础上，支架内外血栓组织可以直接诱发。因此左髂静脉球囊扩张支架植入术后仍需高强度抗凝治疗，以防止支架内继发血栓形成。

目前，这类患者术后抗凝方案为早期肝素抗凝治疗，后期过渡到口服维生素K拮抗剂（华法令）抗凝治疗。华法令作为标准口服抗凝药，抗凝效果确切，价格低廉，但由于该药物治疗窗较窄，患者长期用药需根据凝血功能来调整剂量，造成患者随访管理难度较大、依从性差，治疗效果不确定，甚至引发严重的出血并发症，部分患者自动放弃治疗，血栓复发或加重。新型口服抗凝剂（NOAC）作为口服直接Xa因子抑制剂，具有药物安全性好、治疗方案简单、且不易跟食物药物作用的优点。前期研究表明，利伐沙班在预防抗凝、VTE的抗凝治疗中被证实安全有效，且不需要进行反复的凝血监测。而利伐沙班用于髂静脉狭窄闭塞，以球囊扩张、支架植入术后的辅助抗凝治疗缺乏足够的临床数据验证。

本研究将通过前瞻性、随机对照研究来评估新型口服抗凝剂（NOAC）在左髂静脉球囊扩张支架植入术后抗凝的有效性和安全性。为该疾病诊疗指南及新型口服抗凝药临床治疗适应症探索进行技术准备。

二、具体程序和流程

您的症状体征和影像学检查明确诊断为左髂静脉压迫综合征且行左髂静脉支架植入术，根据纳入和排除标准，如果您符合要求，仔细介绍研究期间存在的风险和要求，签署试验相关之情同意书。纳入的受试者支架植入后随机分为两组，分别予低分子肝素皮下注射继以华法令口服及口服利伐沙班抗凝。抗凝期间需要定期随访，直至术后 24 个月。

三、如果参加研究您需要做什么

积极配合医护人员诊治，严格按照医嘱服用药物和饮食。出院后按照规定时间来门诊随诊。

四、参加本研究可能给您带来的受益

左髂静脉压迫综合征支架植入术后仍需要足量抗凝治疗，常规的低分子肝素+华法令抗凝存在局限性，而利伐沙班抗凝对这类患者的安全性和有效性仍无强有力的临床数据支持。您的参与有助于为这类疾病的治疗提供充实的临床证据。

参加本研究可能给维持您支架植入术后通畅，避免深静脉血栓形成，但是我们不能做出保证。您的参加可能给未来遭受同样痛苦的患者带来益处。

五、参加本研究可能发生的不良反应、风险以及风险防范措施

本研究涉及 2 种抗凝方案 3 种抗凝药物，主要的不良反应是抗凝相关的出血，包括出血性脑卒中、消化道出血、血尿、皮肤粘膜出血、其他内脏出血等。同时存在抗凝不足导致的有症状的复发性的静脉血栓栓塞，支架内血栓形成，支架闭塞等。对于低分子肝素药物来说，还存在皮下注射导致的注射部位的疼痛、皮肤瘙痒、皮下出血及结节等不良反应。口服华法令偶见不良反应有恶心呕吐、腹泻、瘙痒性皮疹，过敏反应及皮肤坏死。大量口服可能出现双侧乳房坏死，微血管病或溶血性贫血以及大范围皮肤坏疽。另外还可能存在目前无法预知风险和不良反应。预防措施包括入研究前严密筛查，建立档案，严密随访，有病情变化即刻处理。医院为通过伦理审查后开展的研究者发起的科研项目购买了保险，若发生与研究相关的严重不良事件，医院将根据相关的法律法规为您争取赔偿。

六、费用情况说明

参加本项临床研究并不增加患者额外的检查，球囊扩张支架植入手术为常规髂静脉受压综合征治疗程序，手术相关花费及住院花费需患者自行承担，随访相关血液化验费需由患者自行承担。术后抗凝药物低分子肝素、华法令及利伐沙班由患者自行承担，对照组（低分子肝素+华法令）6

个月，总花费 2952 元，试验组（利伐沙班 ）6 个月花费 1915 元。患者全部完成随访后可获得 200 元/人的交通费补偿，涉及到试验相关血管无创检查由患者承担（术后随访 6 次，每次检查费 500 元，共计费用 3000 元）。

七、用药指导及随访

建立患者微信群，线上实时指导用药，并及时收集患者情况。

八、参与研究的补偿，包括损伤的赔偿

如果您在研究期间发生了不良事件，该不良事件是否由与本研究的药物和研究方案有关，应由研究者做出判断。确因研究药物和研究方案所致的不良事件并对您造成伤害的，可在您研究所在医院得到积极治疗。但研究者不支付与本研究无关的费用。倘若发生与本研究相关的超出预期的造成严重后果的严重不良事件，且符合医院已投保的临床研究责任险条款，医院将为患者向保险公司争取补偿。

九、替代方案

如果不参加本研究，替代方案是按照常规的抗凝方案如低分子肝素皮下注射抗凝或联合桥接华法林抗凝。您的研究者将会提供适合您的治疗方案，您的研究者也会乐于解释用于治疗您的疾病的其他抗凝方案的可能好处及风险。

十、您个人信息的保密

您的医疗记录（包括研究病历及理化检查报告等）将按规定保存在医院。除研究者、伦理委员会、监查、稽查、药政管理部门等相关人员将被允许查阅您的医疗记录外，其他与研究无关的人员在未得到允许的情况下，无权查阅您的医疗记录。本研究结果的公开报告将不会披露您的个人身份。我们将在允许的范围内，尽一切努力保护您个人医疗资料的隐私。

十一、终止参加研究

是否参加本项研究完全取决于您的自愿。您可以拒绝参加此项研究，或在研究过程中的任何时间无理由退出研究，这都不会影响您和医生的关系，都不会影响对您的医疗或有其他方面利益的损失。

此外，由于以下原因，可能会终止您参与本研究：

1、您不遵从医生的医嘱、依从性不够，不能完成定期随访。

2、您不幸与患其他严重疾病，可直接影响本疾病预后和远期疗效。

3、研究医生认为，终止研究对您的健康和福利最有利。

十二、伦理委员会

本研究已向浙江大学医学院附属第二医院人体研究伦理委员会报告，经委员会的全面审查和包括对受试者的风险评估，并获得了批准。在研究中过程中，**有关伦理和权益事宜可联系浙江大学医学院附属第二医院人体研究伦理委员会，电话：白天 0571-87783759； 晚上（总值班）：13757118366；邮箱地址： HREC2013@126.com**

我确认已阅读并理解了本研究的知情同意书，自愿接受本研究中的治疗方法，并同意将我的医疗数据用于本研究的发表。

受试者签名：__________ 联系方式：______________ 日期：______________

代理人签名：______________与受试者关系______________联系方式_____________ 日期__（如果需要）

见证人（如果需要）：_____________联系方式：______________ 日期：______________

我确认已向患者解释了本研究的详细情况，包括其权利以及可能的受益和风险，并给其一份签署过的知情同意书副本。

研究者签名： _____________

联系方式： _____________（手机） 日期：________________
