# Supplementary material for: An efficacy and safety study of rivaroxaban for the prevention of deep vein thrombosis in patients with left iliac vein compression treated with stent implantation (PLICTS): study protocol for a prospective randomized controlled trial
Source: Trials. 2020 Sep 29;21:811. doi: 10.1186/s13063-020-04742-z (PMC7526216; doi:10.1186/s13063-020-04742-z)
Supplement: Supplementary file 4 — Additional file 4. Supplementary Appendix. [file 13063_2020_4742_MOESM4_ESM.docx]

**Supplementary Appendix**

**The standards for warfarin withdrawal or reduction**

The standards for warfarin withdrawal or reduction are as follows^1^：1) INRs greater than 3.0 but lower than 5.0 and without significant bleeding，lower the dose of warfarin. 2) INRs between 5.0 and 9.0 and without significant bleeding, stop using warfarin and administer vitamin K1 1 to 4mg orally. 3) INRs greater than 9.0 and without significant bleeding, stop using warfarin and administer vitamin K1 3 to 5mg orally. 4) INRs greater than 20.0 and with bleeding, stop using warfarin and administer vitamin K1 10mg and fresh frozen plasma by IV infusion. 5) patients with life-threatening bleeding, stop using warfarin, and administer vitamin K1 10mg by IV infusion supplemented with prothrombin complex by intravenous drip infusion.

**Bleeding Definitions**

International Society on Thrombosis and Haemostasis (ISTH) Bleeding Definitions^2^

Major bleed

• Meets ≥1 of the following criteria:

o Symptomatic bleeding in a critical area or organ, e.g., intracranial, intraspinal, intraocular,

retroperitoneal, intra-articular, or pericardia, or intramuscular with compartment syndrome

o Bleeding associated with a reduction in hemoglobin of ≥2 g/dl (1.24 mmol/l) or leading to

transfusion of ≥2 U blood or packed cells

o Fatal bleed

Minor bleed

• Clinical bleeds that do not fulfill criteria for major bleeds

Clinically relevant nonmajor bleeding event

• A clinically overt bleed that does not meet thecriteria for a major bleed but results in ≥1 of the

following clinical responses:

o A hospital admission

o A physician-guided medical or surgical treatment

o A physician-guided change, interruption (more than omitting 1 dose), or discontinuation of the study drug

**References**

1. Ansell J, Hirsh J, Dalen J, Bussey H, Anderson D, Poller L, et al. Managing oral anticoagulant therapy. Chest. 2001;119:22s-38s.

2. Schulman S, Angerås U, Bergqvist D, Eriksson B, Lassen MR, Fisher W. Definition of major bleeding in clinical investigations of antihemostatic medicinal products in surgical patients. Journal of thrombosis and haemostasis : JTH. 2010;8:202-4.
